# Negotiating mutualism: A locus for exploitation by rhizobia has a broad effect size distribution and context‐dependent effects on legume hosts

**DOI:** 10.1111/jeb.14011

**Published:** 2022-05-04

**Authors:** Camille E. Wendlandt, Miles Roberts, Kyle T. Nguyen, Marion L. Graham, Zoie Lopez, Emily E. Helliwell, Maren L. Friesen, Joel S. Griffitts, Paul Price, Stephanie S. Porter

**Affiliations:** ^1^ 6760 School of Biological Sciences Washington State University Vancouver Washington USA; ^2^ 8759 Biology Department Eastern Michigan University Ypsilanti Michigan USA; ^3^ 6760 Department of Plant Pathology Washington State University Pullman Washington USA; ^4^ 6760 Department of Crop & Soil Sciences Washington State University Pullman Washington USA; ^5^ 6756 Department of Microbiology and Molecular Biology Brigham Young University Provo Utah USA

**Keywords:** cooperation, *hrrP*, legume, peptidase, rhizobia, symbiosis, working balance hypothesis

## Abstract

In mutualisms, variation at genes determining partner fitness provides the raw material upon which coevolutionary selection acts, setting the dynamics and pace of coevolution. However, we know little about variation in the effects of genes that underlie symbiotic fitness in natural mutualist populations. In some species of legumes that form root nodule symbioses with nitrogen‐fixing rhizobial bacteria, hosts secrete nodule‐specific cysteine‐rich (NCR) peptides that cause rhizobia to differentiate in the nodule environment. However, rhizobia can cleave NCR peptides through the expression of genes like the plasmid‐borne *Host range restriction peptidase* (*hrrP*), whose product degrades specific NCR peptides. Although *hrrP* activity can confer host exploitation by depressing host fitness and enhancing symbiont fitness, the effects of *hrrP* on symbiosis phenotypes depend strongly on the genotypes of the interacting partners. However, the effects of *hrrP* have yet to be characterised in a natural population context, so its contribution to variation in wild mutualist populations is unknown. To understand the distribution of effects of *hrrP* in wild rhizobia, we measured mutualism phenotypes conferred by *hrrP* in 12 wild *Ensifer medicae* strains. To evaluate context dependency of *hrrP* effects, we compared *hrrP* effects across two *Medicago polymorpha* host genotypes and across two experimental years for five *E*. *medicae* strains. We show for the first time in a natural population context that *hrrP* has a wide distribution of effect sizes for many mutualism traits, ranging from strongly positive to strongly negative. Furthermore, we show that *hrrP* effect size varies across host genotypes and experiment years, suggesting that researchers should be cautious about extrapolating the role of genes in natural populations from controlled laboratory studies of single genetic variants.

## Graphical abstract

Rhizobia bearing the *hrrP* locus can exploit legumes, but a genetic disruption experiment shows that *hrrP* can also reduce rhizobium fitness (root nodule count) in some genotypic combinations of rhizobia (r1‐r5) and hosts (Legume 1‐2).

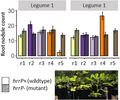

## 1 INTRODUCTION

Mutualisms between hosts and microbes are ubiquitous and play a critical role in spurring evolutionary innovation and powering ecosystem services. However, we still know little about the genetic variants that influence partner fitness in mutualisms, especially compared to antagonisms (Baskett & Schemske, 2015; Stoy et al., 2020). Understanding which genes affect mutualist fitness, how they are transmitted, and when and how they function can help us predict how different mutualism traits will evolve. For instance, genes residing on mobile genetic elements may sweep through microbial populations more rapidly than vertically transmitted genes (Shapiro, 2016), leading to more rapid evolution of mutualism traits. Genes governing early stages of symbiosis, compared to later stages, may increase a symbiont's host range (Radutoiu et al., 2007) and impact its long‐term extinction risk (Koh et al., 2004). Genes with pleiotropic effects may experience stronger evolutionary constraints than genes that only affect single traits (Auge et al., 2019), preventing mutualism traits from reaching optimum values for fitness. Mutualism genes have been uncovered by a variety of methods – including mutant screens, association genetics and quantitative trait locus mapping – that associate mutualism phenotypes with the presence of particular genes or variation among alleles (Burghardt et al., 2017; Gorton et al., 2012; Hu et al., 2020; LaPlante et al., 2021; Piculell et al., 2019; Price et al., 2015; Stanton‐Geddes et al., 2013; Torkamaneh et al., 2020). Increasingly, these methods are equipped to detect loci that exhibit context dependent phenotypes, such as mutualistic partner‐dependent phenotypes (MacPherson et al., 2018; Wang et al., 2018). However, it remains uncertain how much we can extrapolate from highly controlled laboratory studies of single genetic variants to the function of genes in natural populations.

The impact of a gene on rates of phenotypic evolution depends on the effect size distribution of allelic variants, where ‘effect size’ indicates how much an allele changes a particular trait value (Dittmar et al., 2016). Random mutations generate alleles with a range of effect sizes (Bataillon & Bailey, 2014; Kassen & Bataillon, 2006), and the width of the effect size distribution in a population provides the genetic variation upon which natural selection acts (Salvaudon et al., 2008; Simonsen & Stinchcombe, 2014). Wider effect size distributions (i.e. more genetic variance) can produce faster responses to selection (Li, 1967), although narrow effect size distributions can accelerate evolution over short timescales (Briggs & Goldman, 2006). Rates of phenotypic evolution will also depend on the amount of context dependency in the effect size of a candidate gene. Context dependency exists when the effect size of a gene varies with environmental conditions (i.e. phenotypic plasticity) or genotypes at other loci (i.e. epistasis; Remold & Lenski, 2004). High context dependency can alter selection on an allele by limiting the contexts in which it confers effects on fitness. Thus, context dependency can prevent an allele from sweeping through a population, even when selection is strong (Chevin, 2019; Höllinger et al., 2019), or conversely, context dependency can accelerate evolution towards a phenotypic optimum (Borenstein et al., 2006). Given the strong impacts that the distribution and context dependency of effect size can have on the tempo of evolution, it is critical to study these parameters for genes important in mutualisms.

The legume‐rhizobium symbiosis is a globally important mutualism that shapes the ecology of wild plant communities (van der Heijden et al., 2006) and provides much of the nitrogen needed in agriculture (Goyal et al., 2021). The symbiosis is initiated when soil‐dwelling rhizobial bacteria infect the roots of leguminous plants, forming nodules in which they fix atmospheric nitrogen into a form plants can use for growth (Poole et al., 2018). In a subset of legumes, differentiation of rhizobia into their nitrogen‐fixing form is accomplished by host secretion of nodule‐specific cysteine‐rich (NCR) peptides, which target the organelle‐like structures in which rhizobia are sequestered and trigger rhizobia to undergo partial membrane permeabilisation, genome duplication and loss of reproductive viability inside the nodule (Alunni & Gourion, 2016; Ledermann et al., 2021). The genome of the model legume *Medicago truncatula* encodes an abundant and diverse family of NCR peptides (Montiel et al., 2017), and plant accessions vary substantially in expression levels of individual NCR peptide genes (Nallu et al., 2014). The role of NCR peptides in rhizobial adaptation to the nodule environment is complex, and most have not been functionally studied. On one hand, some NCR peptides have antimicrobial activity and can kill rhizobia in the nodule, depending on the plant genomic background (Yang et al., 2017). On the other hand, certain NCR peptides are required for rhizobia to persist in nodules, and rhizobia die if hosts fail to express these peptides (Horváth et al., 2015; Kim et al., 2015).

Far from being passive recipients of NCR peptide cues, rhizobia can curtail this signalling mechanism by producing peptidases that degrade specific NCR peptides (Benedict et al., 2021; Price et al., 2015). One such agent is Host range restriction peptidase (HrrP), a plasmid‐encoded peptidase discovered in *Ensifer meliloti*, the rhizobial symbiont of *M*. *truncatula* (Crook et al., 2012; Price et al., 2015). *hrrP* was uncovered in a mutant screen for rhizobia that gain compatibility with novel hosts: disruption of the *hrrP* locus allows *E*. *meliloti* to fix nitrogen on a host with which it is otherwise incompatible. In some cases, *hrrP*‐expressing rhizobia can avoid differentiating and fixing nitrogen inside nodules, which decreases plant fitness but increases rhizobium fitness (Price et al., 2015). However, the effect of *hrrP* on host and rhizobium fitness is dependent on host genotype and strain genomic background (Price et al., 2015), with some host genotypes appearing totally resistant to the activity of *hrrP* (i.e. experiencing normal nitrogen fixation from *hrrP*‐expressing rhizobia), and with some strain genotypes exhibiting different phenotypes even when bearing the same *hrrP* allele (Price et al., 2015). The context dependency of the effects of NCR peptides and rhizobium peptidases on host and symbiont fitness is captured by the ‘working balance’ hypothesis of peptidase‐NCR peptide dynamics (Pan & Wang, 2017), which predicts that rhizobia and hosts benefit from moderate net NCR peptide levels, but show extreme phenotypes when NCR peptides are excessively low or high. Since *hrrP* serves as a means for rhizobia to tune their host's level of NCR peptide production, the effect size of *hrrP* is predicted to vary among hosts to the extent that hosts vary in NCR peptide expression, consistent with (Price et al., 2015). Sequence variation in the *hrrP* locus could also contribute to variation in *hrrP* effect size, although only one *hrrP* allele has been empirically studied to date (Price et al., 2015).

Here, we investigate the distribution and context dependency of *hrrP* effects on mutualism outcomes. We used a wild collection of the rhizobium *E*. *medicae*, which can nodulate several *Medicago* species including *M*. *polymorpha* and *M*. *truncatula* (Denton et al., 2007). In a previous PCR screen, 12.5% of the *E*. *medicae* strain collection was found to bear *hrrP* (i.e., *hrrP*+ strains), with the remainder lacking this locus (i.e. *hrrP*‐ strains; Wendlandt et al., 2021). We performed targeted gene disruptions in 12 *hrrP*+ *E*. *medicae* strains to generate *hrrP*‐ knockout mutants, and measured *hrrP* effect size for each strain as the relative difference in trait values of *hrrP*+ and *hrrP*‐ strains. Because we used wild *E*. *medicae* strains, this measure of *hrrP* effect size could be influenced by variation among strains in *hrrP* allelic identity (affecting its specificity and catalytic activity for targeted NCR peptides), *hrrP* expression level (due to variation in promoter sequence or other modifiers of gene expression), and other loci with epistatic effects. This measure of *hrrP* effect size is intentionally broad in scope to capture natural phenotypic consequences of disruption to this locus in nature, making it an ecologically relevant way to understand *hrrP* effect size. To assess how variation among rhizobia strains, plant host genotypes, and environments alters the impact of *hrrP* on mutualism outcomes, we asked: (1) Do *hrrP* effect sizes differ among strains of wild *E*. *medicae*? And, do *hrrP* effect sizes show context dependency across (2) different *M*. *polymorpha* plant host genotypes and (3) different experiment years?

## 2 METHODS

### 2.1 Experimental design

We performed two greenhouse experiments in which sterile seedlings of *Medicago polymorpha* were inoculated with single strains of *Ensifer medicae* or cell‐free media (control inoculations). We measured proxies of plant and rhizobium fitness after approximately 6 weeks of growth. All experiments were performed in a greenhouse at Washington State University Vancouver (45.7328054° N, 122.635967° W). The Knockout Experiment included one *M*. *polymorpha* plant host genotype inoculated with 12 *hrrP*‐bearing (*hrrP*+) strains and 12 knockout (*hrrP*‐) mutant strain derivatives (Table 1). Strain treatments were replicated over 17 blocks and each block included one uninoculated control plant (425 plants total). The Knockout Experiment tests how *hrrP* effect size varies among *E*. *medicae* strains (question 1). The G × G Knockout Experiment includes two *M*. *polymorpha* plant host genotypes inoculated with 5 *hrrP*‐bearing (*hrrP*+) strains and 5 knockout (*hrrP*‐) mutant strain derivatives (Table 1). Strain × host treatments were replicated over 15 blocks and each block included one uninoculated control plant per plant host genotype (330 plants total). The G × G Knockout Experiment tests for context dependency of *hrrP* effect size between host genotypes (question 2), and when compared to data from the Knockout Experiment, for context dependency between experiment years (question 3).

**TABLE 1:** *Medicago polymorpha*and *Ensifer medicae* genotypes used in each greenhouse experiment

Genotype	Knockout Experiment (2018)	G × G Knockout Experiment (2019)	GenBank Accession
*M. polymorpha*			
MEL		X	
RTM	X	X	
*E. medicae*			
AZN131	WT, KO		MW417466
AZN234	WT, KO	WT, KO	MW417464
DCR341	WT, KO	WT, KO	MW417456
PEA63	WT, KO	WT, KO	MW417438
PEA143	WT, KO	WT, KO	MW417441
RTM196	WT, KO	WT, KO	MW417435
RTM371	WT, KO		MW417431
RTM372	WT, KO		MW417430
RTM373	WT, KO		MW417429
RTM376	WT, KO		MW417428
STA354	WT, KO		MW417425
STA355	WT, KO		MW417424

Note

For *M*. *polymorpha* plant hosts, ‘X’ indicates that the plant host genotype was used. For *E*. *medicae* rhizobia, ‘WT’ indicates that the wild‐type *hrrP*+ strain was used, and “KO” indicates that the knockout *hrrP*− strain was used. GenBank accessions refer to *hrrP* sequences for each *E*. *medicae* strain.

### 2.2 Rhizobia strains and inocula preparation

In the Knockout Experiment, we used 12 *E*. *medicae* strains (Table 1) genotyped as *hrrP*+ by Wendlandt et al. (2021). These 12 strains span the genetic diversity uncovered for *hrrP* by Wendlandt et al. (2021) and represent 6 unique *hrrP* sequences for the partial coding region for which sequence data are available (Table S2). We generated one *hrrP*‐ knockout mutant strain from each of the 12 *E*. *medicae* strains using homologous recombination insertional mutagenesis. Briefly, a 3 kb non‐replicative plasmid encoding a neomycin resistance gene was inserted into the *hrrP* coding region, and the presence of the insert was verified by testing for neomycin resistance and performing PCR with primers whose product spans the gene‐insert junction (Price et al., 2015). Previous tests of mutants made in this way found no pleiotropic effects of neomycin insertion (Paul Price, pers comm). In the G × G Knockout Experiment, we used five of the wild‐type *hrrP*+ strains used previously as well as their knockout *hrrP*‐ derivatives (Table 1).

We prepared rhizobial inocula for the greenhouse experiments by streaking frozen glycerol stocks of each wild‐type and knockout strain onto tryptone yeast (TY) agar plates and incubating until single colonies formed. Before preparing inocula, we confirmed that *hrrP* could be PCR‐amplified from each wild‐type strain in the upcoming experiment. Single colonies were then used to inoculate 1 ml of aliquots of TY broth, which were incubated at 30°C and 300 rpm for 3 days. Two hundred and fifty microlitres of the 1‐mL culture was used to inoculate 4.75 ml of TY broth, which was incubated at 28°C and 300 rpm for 2 days. The OD_600_ was measured for each culture to estimate the number of colony‐forming units (CFUs) using a conversion factor of CFU ml^−1^ = 5.8 × 10^7^ × OD_600_. Cells were pelleted, separated from supernatant and resuspended in 0.1X TY broth to concentrations of approximately 10^6^ CFU ml^−1^. Following Heath and Tiffin (2009), we assumed that the relationship between OD_600_ and CFU was approximately consistent across strains. Since our main goal was to inoculate plants with enough rhizobia (10^6^ CFU) that nodule formation would not be limited by the number of rhizobia present, moderate fluctuations in the relationship between OD_600_ and CFU among strains should have weak impacts on our findings. See Table S1 for specific methods used in each experiment.

### 2.3 Plant host genotypes and growth conditions

We generated all seeds in a common garden in greenhouse conditions to minimise maternal effects. In the Knockout Experiment, we used one *M*. *polymorpha* genotype (RTM; Table 1). Plant host genotypes were named for the populations from which they were isolated; thus, the RTM host was sympatric to all the *E*. *medicae* strains having ‘RTM’ in their name (Wendlandt et al., 2021). In the G × G Knockout Experiment, we used two *M*. *polymorpha* genotypes (RTM, MEL; Table 1). For each greenhouse experiment, seeds were scarified on sandpaper, stratified at 4°C for approximately one week, surface‐sterilised by exposure to chlorine gas for 6 h and planted into autoclaved 158‐ml containers filled with a 1:1 mix of Sungro Sunshine Mix #1 and sand. Plants were mist‐irrigated for 10 min twice daily throughout germination and until the end of the experiment. Two weeks post sowing, seedlings were inoculated with a rhizobium cell suspension or cell‐free control, pipetted at the base of the plants (900 µl per plant for the Knockout Experiment; 450 µl per plant for the G × G Knockout Experiment). A few days after inoculation, autoclaved sand was added in a 5‐mm deep layer around each seedling to minimise cross‐contamination of treatments. Plants were fertilised with 2 ml of 0.5× Fahreus solution (Vincent, 1970) containing 500 µM of NH_4_NO_3_, beginning the week after inoculation. Plants were fertilised weekly for a total of five times throughout the experiment; see Table S1 for details.

### 2.4 Measuring plant traits and *hrrP* effect size

For the Knockout Experiment, we counted the number of trifoliate leaves on plants just prior to harvest. For both experiments, plants were harvested starting 39–40 days post inoculation, proceeding by experimental block to avoid a treatment bias in date of harvest. Shoots were clipped from roots, dried in a 60°C oven and weighed. Roots were washed free of substrate in a sieve and stored on ice. We excised one nodule from each nodulated plant for measuring the number of CFUs per nodule following the culturing protocol in Wendlandt et al. (2021). We inadvertently used slightly different nodule selection criteria for the different experiments: in the Knockout Experiment, we selected a nodule representative in size of most nodules on the plant, and in the G × G Knockout Experiment, we selected the largest, reddest nodule on the plant. However, because nodule selection criteria were consistent within each experiment, and our primary findings are effect sizes derived from measurements within each experiment, our comparisons of effect sizes between experiments reflect biological differences in how treatments impacted CFU per nodule. CFU per nodule is positively related to nodule size in the *Medicago*‐*Ensifer* system (Porter & Simms, 2014) and reflects the fitness benefit for a single rhizobium cell founding a nodule. Roots were frozen and later thawed to count the total number of nodules per plant.

In total, we measured up to six traits from each experiment. As proxies of plant fitness, we measured leaf count, dry shoot mass and dry shoot mass per nodule (reflecting the balance of benefits to plants versus rhizobia). As proxies of rhizobium fitness, we measured total nodule count, nodule size and CFU per nodule (the latter was log‐transformed before analysis). Pairwise correlation coefficients for these responses are reported in Table S3. For each trait, we calculated *hrrP* effect size using pairs of plants of the same genotype and from the same block that were inoculated with wild‐type (*hrrP*+) or knockout (*hrrP*−) versions of the same *E*. *medicae* strain:

$$hrrP\text{effect}\;\text{size}=\frac{{\text{Trait}}_{\text{WT}}‐{\text{Trait}}_{\text{KO}}}{{\text{Trait}}_{\text{KO}}}$$

We scaled the trait differences to the trait value of the knockout mutant strain so that *hrrP* effect size reflects the consequence of an *hrrP*− strain gaining *hrrP*. Thus, *hrrP* effect sizes can range from negative to positive, based on whether *hrrP* decreases or increases the trait value, respectively.

### 2.5 Statistical analysis

We analysed data using general linear mixed models implemented with lme4 v. 1.1–21 in R v. 3.5.2 (R Core Team, 2018). Models used Gaussian errors and residuals were checked with DHARMa v. 0.2.7 (Hartig, 2019). We used likelihood ratio tests to assess significance of all fixed effects. All models included a random effect of block. To test for differences in *hrrP* effect size among *E*. *medicae* strains (question 1), we modelled *hrrP* effect size with a fixed effect of Strain using data from the Knockout Experiment (Model 1, Table 2). We examined confidence intervals for parameter estimates of *hrrP* effect size to infer whether *hrrP* had neutral or nonzero effect sizes (i.e. effect sizes with confidence intervals not overlapping zero) for each *E*. *medicae* strain. To test for context dependency of *hrrP* effect size between different host genotypes (question 2), we modelled *hrrP* effect size with fixed effects of Host, Strain and the Host:Strain interaction using data from the G × G Knockout Experiment (Model 2, Table 2). To test for context dependency of *hrrP* effect size between experiment years (question 3), we pooled data from the Knockout Experiment and the G × G Knockout Experiment and modelled *hrrP* effect size with fixed effects of Year, Strain and the Year:Strain interaction (Model 3, Table 2). We considered *hrrP* effects ‘consistent’ between plant host genotypes (or between experiment years) if effect sizes had the same sign (negative, neutral or positive) on both plant host genotypes (or in both years). Inconsistent *hrrP* effects between host genotypes and experiment years were interpreted as evidence of context dependency.

**TABLE 2:** Likelihood ratio test χ^2^ values for GLMMs analysing variation in *hrrP* effect size for several traits of *M*. *polymorpha* plant hosts inoculated with *E*. *medicae* rhizobia

Model, Term	Leaf count	Shoot mass	Shoot mass per nodule	Nodule count	Log(CFU per nodule)
*Model 1*	*n*= 202	*n*= 202	*n*= 197	*n*= 196	*n*= 185
Strain	39.16***	57.51***	61.36***	11.65	36.47**
*Model 2*		*n*= 145	*n*= 116	*n*= 116	*n*= 108
Host	na	3.58†	0.04	1.66	1.02
Strain	na	83.51***	8.94*	14.29*	0.32
Host:Strain	na	3.83	9.40*	17.47**	2.06
*Model 3*		*n*= 156	*n*= 124	*n*= 123	*n*= 116
Year	na	11.02**	0.01	0.05	4.64*
Strain	na	82.93***	7.61†	13.61*	0.81
Year:Strain	na	14.70*	13.18*	15.35*	1.20

Note

**FIGURE 1:**
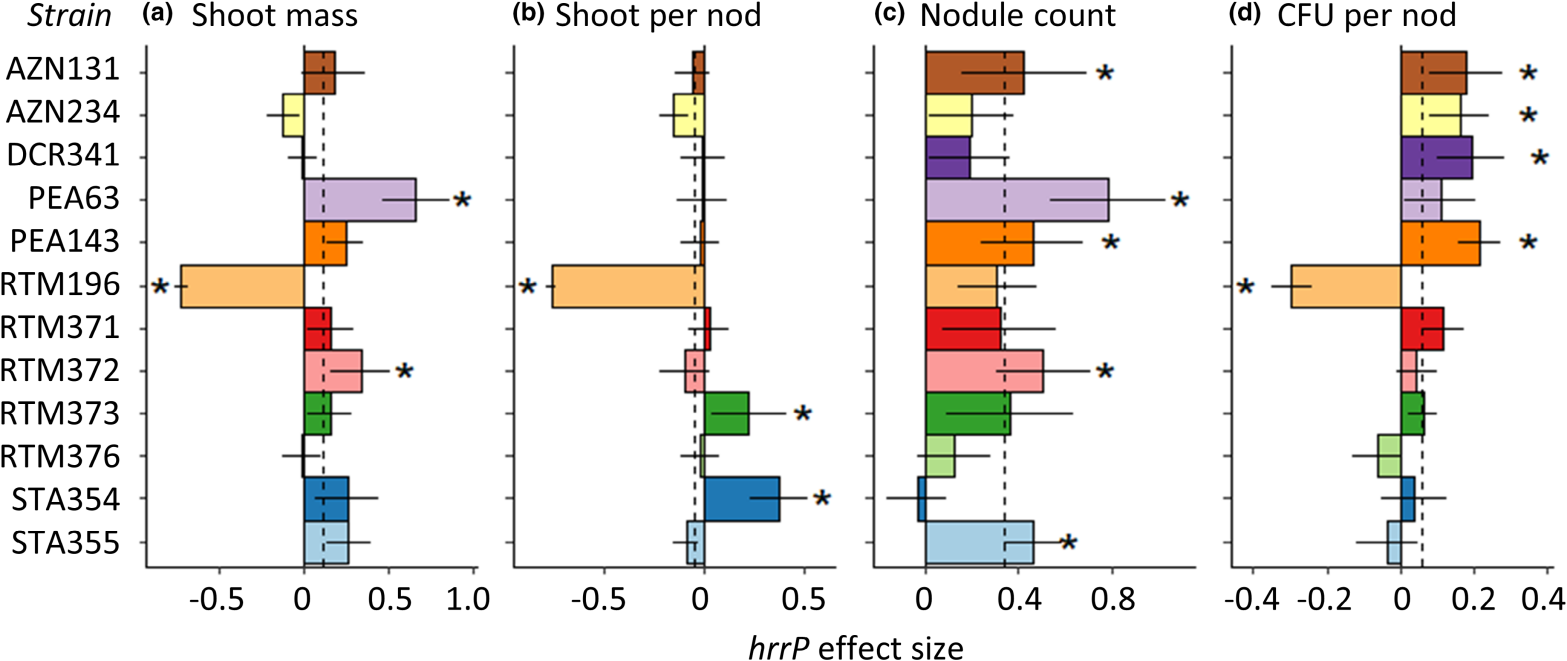
Effect size of *hrrP* on mutualism outcomes varies among *Ensifer medicae* strains. *hrrP* effect size indicates the proportional change in a trait value due to the presence of *hrrP*, using comparisons of wild‐type *hrrP*+ and knockout *hrrP*− mutant strains in the Knockout Experiment (Model 1). Asterisks indicate parameter estimates of *hrrP* effect size for which the 95% confidence interval did not include zero. The dashed vertical line indicates the mean *hrrP* effect size across all 12 *E*. *medicae* strains. Bars represent ± 1 standard error. One outlier was excluded from the nodule count data

**FIGURE 2:**
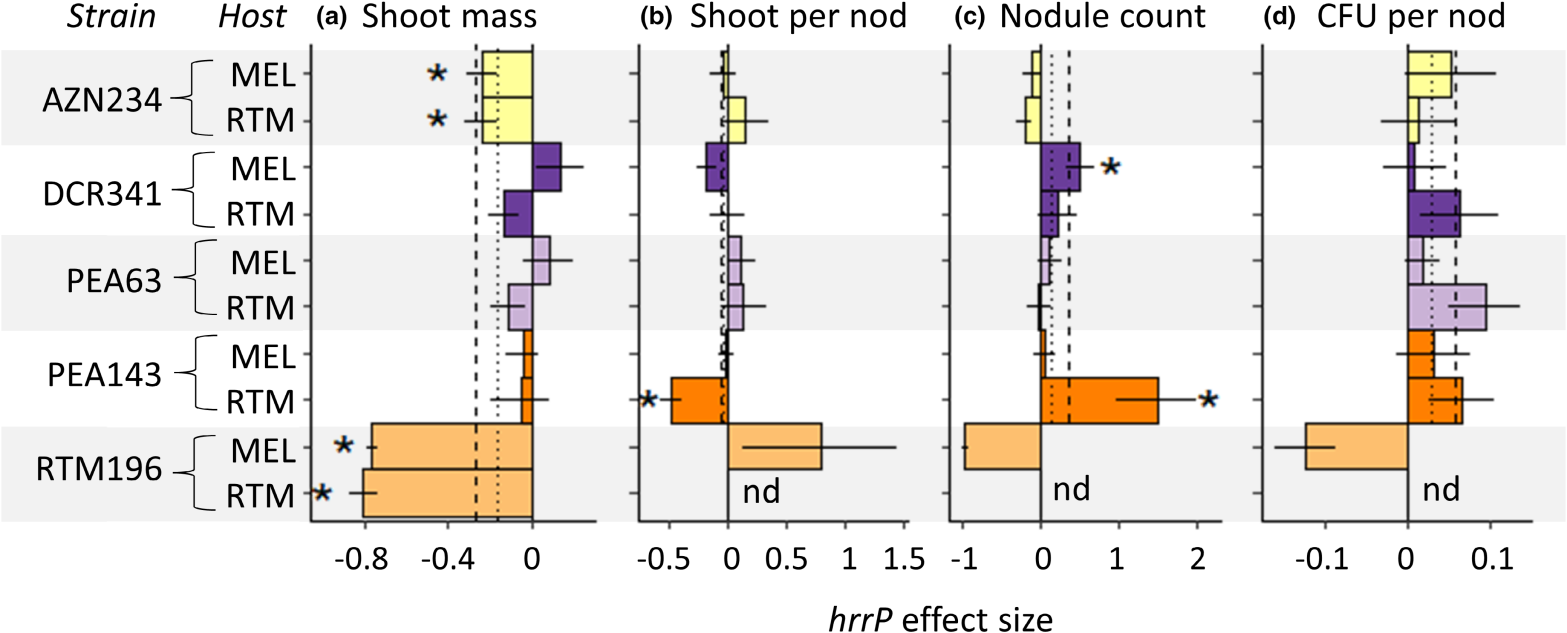
Partner genotype context dependence: *hrrP* effect size differs among *Medicago polymorpha* plant host genotypes. *hrrP* effect size indicates the proportional change in a trait value due to the presence of *hrrP*, using comparisons of wild‐type *hrrP*+ and knockout *hrrP*− mutant strains. Effect sizes were measured in the G × G Experiment (Model 2). Asterisks indicate parameter estimates of *hrrP* effect size for which the 95% confidence interval did not include zero. Vertical lines indicate the mean *hrrP* effect size across all *E*. *medicae* strains for *M*. *polymorpha* RTM (dashed) and *M*. *polymorpha* MEL (dotted). Since the wild‐type *E*. *medicae* RTM196 strain did not form nodules on *M*. *polymorpha* RTM in 2019, we did not include this strain in analyses of shoot mass per nodule, nodule count, or logCFU per nodule; in panels B‐D, we did not place asterisks for the *E*. *medicae* RTM196 or use data from this strain for calculating mean effect size for each year. Bars represent ± 1 standard error. nd = no data

**FIGURE 3:**
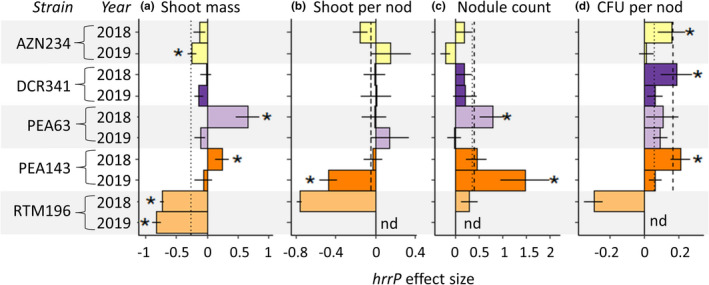
Environmental context dependence: *hrrP* effect size differs across experiment years. *hrrP* effect size indicates the proportional change in a trait value due to the presence of *hrrP*, using comparisons of wild‐type *hrrP*+ and knockout *hrrP*− mutant strains. Effect sizes were measured on *Medicago polymorpha* RTM in the pooled Knockout Experiment and G × G Knockout Experiment (Model 3). Asterisks indicate parameter estimates of *hrrP* effect size for which the 95% confidence interval did not include zero. Vertical lines indicate the mean *hrrP* effect size across all strains for 2018 (dashed) and 2019 (dotted). Since the wild‐type *E*. *medicae* RTM196 strain did not form nodules on *M*. *polymorpha* RTM in 2019, we did not include this strain in analyses of shoot mass per nodule, nodule count, or logCFU per nodule; in panels B–D, we did not place asterisks for the *E*. *medicae* RTM196 strain or use data from this strain for calculating mean effect size for each year. Bars represent ± 1 standard error. nd = no data

Model 1 tested for variation among strains in *hrrP* effect size among 12 *E*. *medicae* strains on one *M*. *polymorpha* host genotype (Knockout Experiment, Figures 1 and S2). Model 2 tested for effects of host genotype on *hrrP* effect size using 5 *E*. *medicae* strains and 2 *M*. *polymorpha* host genotypes (G × G Knockout Experiment, Figure 2). Model 3 tested for effects of experiment year on *hrrP* effect size using 5 *E*. *medicae* strains and one *M*. *polymorpha* host genotype (pooled Knockout Experiment and G × G Knockout Experiment, Figure 3). For each model and response variable, *n* indicates the number of plants used in the analysis. ***p < 0.0001, **p < 0.001, *p < 0.05, †*p* < 0.10.

## 3 RESULTS

### 3.1 The effect size of *hrrP* varies among strains of wild rhizobia

We found that *hrrP* had positive effects on many proxies of plant host and rhizobium fitness. On average, *hrrP* increased leaf count by 8% (Figure S1), increased shoot mass by 11% (Figure 1a), decreased shoot mass per nodule by 6% (Figure 1b), increased nodule count by 34% (Figure 1c), and increased logCFU per nodule by 7% (Figure 1d). However, *E*. *medicae* strains varied significantly in *hrrP* effect size for leaf count, shoot mass, shoot mass per nodule, and logCFU per nodule (Model 1: ‘Strain’; Table 2). At one extreme, in *E*. *medicae* RTM196, *hrrP* decreased leaf count, shoot mass, shoot mass per nodule and CFU per nodule (Figures 1 and S1). However, *hrrP* also showed positive effects on leaf count (for *E*. *medicae* PEA63 and RTM372), shoot mass (for *E*. *medicae* PEA63 and RTM372), shoot mass per nodule (for *E*. *medicae* RTM373 and STA354), nodule count (for *E*. *medicae* AZN131, PEA63, PEA143, RTM372 and STA355), and CFU per nodule (for *E*. *medicae* AZN131, AZN234, DCR341 and PEA143; Figures 1 and S1). Out of 60 strain × trait measurements of *hrrP* effect size (12 strains ×5 traits), 4 measurements of *hrrP* effect size were negative, 41 were neutral and 15 were positive (Figures 1 and S1).

### 3.2 The effect size of *hrrP* varies among plant host genotypes

Mean *hrrP* effect sizes were similar on average for the two plant host genotypes in the G × G Knockout Experiment (Model 2: ‘Host’; Table 2). However, the effects of *E*. *medicae* strain on *hrrP* effect size varied between plant host genotypes for shoot mass per nodule and nodule count (Model 2: ‘Host:Strain’; Table 2). Between plant host genotypes, *hrrP* had inconsistent effects on shoot mass per nodule for one strain (*E*. *medicae* PEA143) and nodule count for two strains (*E*. *medicae* DCR341 and PEA143; Figure 3). Surprisingly, wild‐type *E*. *medicae* RTM196 failed to form any nodules on *M*. *polymorpha* RTM plant hosts in 2019. We did not include this strain in analyses of the remaining (nodule‐based) traits, although we acknowledge the failure to nodulate shows extreme context dependency between plant host genotypes. Out of 17 strain × trait measurements of *hrrP* effect size on each plant host genotype, 14 measurements of *hrrP* effect size were consistent and 3 were inconsistent between plant host genotypes (Figure 2).

### 3.3 The effect size of *hrrP* varies among experiment years

For the 5 *E*. *medicae* strains tested in two experimental years, mean *hrrP* effect size tended to be smaller or more negative in 2019 compared to 2018 (Model 3: “Year”; Table 2). On average, *hrrP* increased shoot mass by 1% in 2018 but decreased shoot mass by 27% in 2019 (Figure 3a), and *hrrP* increased logCFU per nodule by 17% in 2018 and by 6% in 2019 (Figure 3d). Since we selected nodules for CFU estimation using slightly different criteria in each year, the difference in *hrrP* effects on logCFU in each year could be partly due to this methodological difference. The effect of *E*. *medicae* strain on *hrrP* effect size varied between experiment years for shoot mass, shoot mass per nodule and nodule count (Model 3: ‘Year:Strain’; Table 2). *hrrP* had inconsistent effects on shoot mass between years for three strains (*E*. *medicae* AZN234, PEA63 and PEA143; Figure 3a). Since the wild‐type *E*. *medicae* RTM196 failed to form nodules on *M*. *polymorpha* RTM plant hosts in 2019, we did not include this strain in analyses of the remaining (nodule‐based) traits. Between experiment years, *hrrP* also had inconsistent effects on shoot mass per nodule for one strain (*E*. *medicae* PEA143; Figure 3b), nodule count for two strains (*E*. *medicae* PEA63 and PEA143; Figure 3c), and CFU per nodule for three strains (*E*. *medicae* AZN234, DCR341 and PEA143; Figure 3d). Out of 17 strain × trait measurements of *hrrP* effect size in each year, 8 measurements of *hrrP* effect size were consistent and 9 were inconsistent between experiment years (Figure 3).

## 4 DISCUSSION

Predicting the evolutionary dynamics of genes involved in mutualism requires that we understand how these genes contribute to standing genetic variation in natural populations and the degree of context dependency of their phenotypic effects. However, this information is lacking for most loci impacting fitness in mutualism, particularly loci that show large effects in controlled laboratory experiments. Our study reveals that the effects of *hrrP*, a horizontally transmitted, plasmid‐borne locus that can have major effects on the fitness of both mutualist partners, are highly genetically and environmentally context dependent in a set of wild rhizobia strains. We find that: (1) the effect size of *hrrP* on symbiotic partner fitness can differ in sign and magnitude among wild rhizobia strains, and that *hrrP* effect size shows context dependency between (2) different host genotypes and (3) different experiment years. The wide effect size distribution and significant context dependency we reveal for *hrrP* effects suggest that the evolutionary impacts of candidate mutualism loci may be complex in natural mutualist populations.

### 4.1 Variation in *hrrP* effect size among wild *E. medicae* strains

In contrast to previous findings that *hrrP* can improve the performance of rhizobia at the expense of plant hosts (Price et al., 2015; Wendlandt et al., 2021), we show that, on average, the presence of *hrrP* in a rhizobium strain's genome has positive effects on both plant host and rhizobium performance. Our findings broadly align with the working balance hypothesis of peptidase‐NCR peptide activity, which predicts that complete suppression of HrrP activity (corresponding to our knockout mutants) harms both plant hosts and rhizobia by allowing host‐derived NCR peptides to over‐differentiate rhizobia to the point that they are incapable of nitrogen fixation (Pan & Wang, 2017). Instead, moderate HrrP activity is predicted to optimise the fitness of both partners. Consistent with this hypothesis, we saw positive average effects of *hrrP* on fitness metrics for both plant hosts (i.e. leaf count, shoot mass) and rhizobia (i.e. nodule count, logCFU per nodule). Although this contrasts with previous work on the B800 *hrrP* allele, which decreases plant fitness (Price et al., 2015), we identified one strain in which *hrrP* had B800‐like effects on plant fitness (i.e. *E*. *medicae* RTM196), showing that the B800 *hrrP* allele phenotype falls within the range of what we uncovered in our wider survey of strains. We also saw that *hrrP* increased nodule count more than shoot mass, such that *hrrP* decreased shoot mass per nodule and shifted the balance of symbiotic benefits towards rhizobia. This could be a subtle form of exploitation within the constraints of the working balance hypothesis, whereby *hrrP* evolves to increase benefits to rhizobia more than it increases benefits to plant hosts (Klein et al., 2022).

Although previous research uncovered a single *hrrP* allele of large effect (Price et al., 2015), we find that *hrrP* has small or neutral effects on symbiotic traits in many *E*. *medicae* strains. This finding suggests that researchers should be cautious about interpreting the role large‐effect genes will have in nature until more variants of that gene have been studied. Although in certain contexts, *hrrP* can be a strong driver of variation in host and symbiont fitness, our data suggest that large‐effect *hrrP* alleles such as the B800 allele (Crook et al., 2012; Price et al., 2015) may not be common in natural populations, where many *hrrP* alleles have smaller effects on symbiosis traits. One mechanism that could favour small‐effect *hrrP* loci is suggested by the working balance hypothesis, which predicts that hosts and symbionts experience selection towards similar net levels of NCR peptide activity and thus could experience fitness alignment (Friesen, 2012), despite the antagonistic effects of peptidases on host peptides. Since net levels of NCR activity are epistatically determined by both rhizobium *hrrP* and host NCR peptide genes, selection on individual *hrrP* loci would vary depending on the host's complement of NCR peptide genes. Under such variable selection, evolution could favour *hrrP* alleles of small effect, since small‐effect alleles would be less likely to move net NCR activity into fitness valleys for rhizobia. Rhizobia with large‐effect *hrrP* alleles may only occasionally encounter hosts with the exact NCR peptide expression level that results in high fitness for rhizobia, making large‐effect *hrrP* alleles less favourable on average than small‐effect alleles.

The evolution of mutualistic traits could also be driven by the complete gain or loss of *hrrP* by horizontal gene transfer, since *hrrP* is located on a transmissible plasmid (Crook et al., 2012). Across Europe and North America, *hrrP* loci are present in only 13% of *E*. *medicae* strains and are only detectable in 56% of *E*. *medicae* populations (Wendlandt et al., 2021), consistent with a lack of strong fitness benefit of *hrrP* for rhizobium or host fitness. The fact that naturally occurring *hrrP*+ and *hrrP*− rhizobia have only slightly different mean phenotypic effects on hosts (Wendlandt et al., 2021) could reflect a situation in which *hrrP*‐ strains phenotypically resemble *hrrP*+ strains for which *hrrP* has a nearly neutral effect size. If large‐effect *hrrP* alleles arose and conferred a fitness benefit to rhizobia, horizontal gene transfer could accelerate their sweep through rhizobium populations, reducing *hrrP* genetic diversity and increasing the average *hrrP* effect size within populations where large‐effect alleles arise. Consistent with this idea, the strains with the largest *hrrP* effect sizes in our study (*E*. *medicae* RTM196 and PEA63) were isolated from rhizobium populations where *hrrP* is at relatively high incidence (present in 53% and 25% of strains, respectively; Wendlandt et al., 2021). Thus, it is possible that *hrrP* alleles of large effect arose in those populations and are spreading through horizontal gene transfer, although we have evidence that *hrrP* reduces, rather than enhances, fitness for *E*. *medicae* RTM196. It would be valuable for future studies to test whether high frequencies of *hrrP*+ rhizobia occur in populations where plant hosts have high NCR peptide expression (i.e. trait matching; Zangerl & Berenbaum, 2003), which we would predict if *hrrP* and NCR peptide genes are coevolving.

### 4.2 Context dependency of *hrrP* effects

We find *hrrP* effects to be highly context dependent across different *M*. *polymorpha* host genotypes and experimental years. For instance, in *E*. *medicae* PEA63, *hrrP* increased nodule count for *M*. *polymorpha* RTM in 2019 but had no effect on this trait in 2018. Another instance of context dependency involves *E*. *medicae* RTM196, where *hrrP* had no effect on nodule count in 2018 but reduced nodule count to zero in 2019. Although this could be the result of a methodological error during inoculation in 2019, there is precedent for rhizobia to sometimes fail to nodulate plants, potentially due to lower compatibility of the strain‐host combination (Torres‐Martinez et al., 2021). Repeating this inoculation treatment would help distinguish between these possibilities. Furthermore, the finding that *hrrP* effect size diverged strongly between *E*. *medicae* RTM196 and the other *E*. *medicae* RTM strains (RTM371, RTM372, RTM373, and RTM376), even though these strains shared the same partial *hrrP* sequence, supports results from Price et al. (2015) in which *hrrP* effects depend on the action of other loci in the strain genome. Broadly, the context dependency of *hrrP* effects could arise from variation in the expression levels of *hrrP* and/or the NCR peptides degraded by HrrP. Thus, a particular *E*. *medicae* strain could display a large *hrrP* effect size with a plant host that had moderate NCR peptide expression, but a smaller effect size on a host with high NCR peptide expression, and a strain acquiring a novel *hrrP* allele through horizontal gene transfer could express *hrrP* to a different degree than the *hrrP*+ donor strain, due to epistatic interactions between the strain genome and *hrrP*. Furthermore, *hrrP* and NCR peptide expression could also vary depending on the physiological state or developmental stage of the plant host, contributing further to context dependency of *hrrP* effect size.

Finally, the context dependency of *hrrP* effects could drive complex coevolutionary dynamics in wild rhizobia populations. Host‐mediated selection on *hrrP*‐bearing strains would be predicted to differ based on whether strains are interacting with a host on which *hrrP* increases cooperation, versus a host on which *hrrP* has no effect on cooperation. Controlling *hrrP* allelic identity and testing for effects of *hrrP* expression level on strain and host fitness would be a useful next step for exploring this gene's role in coevolution of plants and rhizobia. If there is coevolution between the expression levels of *hrrP* and expression levels of the NCR peptides that HrrP targets for degradation, we would expect the fitness of rhizobia with a particular *hrrP* expression level to depend on mean plant expression of the targeted NCR peptide, and for the fitness of plants with a particular expression level of the targeted NCR peptide to depend on mean *hrrP* expression by rhizobia (following Gomulkiewicz et al., 2007).

### 4.3 Conclusions

Both mutualists and pathogens can have large fitness effects on their hosts, but we generally know more about the genes underlying pathogen interactions than the genes underlying mutualistic interactions. Since mutualisms differ from antagonisms in that partners coordinate to exchange a service or resource, the genetic basis of interaction outcomes may be fundamentally more complex for mutualisms than for antagonisms (Stoy et al., 2020). In line with this anticipated complexity, we show that *hrrP* from a panel of wild *E*. *medicae* strains has a wide range of effect sizes on mutualism outcomes for legumes and rhizobia, and that these effect sizes are highly context dependent. These findings are consistent with the “working balance” model of peptidase‐NCR peptide activity, in which the fitness value of *hrrP* depends on host levels of NCR peptide production. The high context dependency of *hrrP* effects could also contribute to the evolutionary stability of mutualism by preventing genes of large effect from sweeping through symbiont populations. Furthermore, we highlight the importance of measuring effect sizes and context dependency of multiple variants of candidate mutualism genes to understand their probable role in the evolution of wild populations.

## CONFLICTS OF INTEREST

The authors declare they have no conflicts of interest.

## AUTHOR CONTRIBUTIONS

MLG, PP, and JSG prepared *hrrP*‐ mutant *E*. *medicae* strains. SSP, MLF, EEH, and ZL designed the experiment. EEH, ZL, MR, KTN, and CEW performed the experiment and collected data. CEW and SSP analysed the data and wrote the manuscript.

### PEER REVIEW

The peer review history for this article is available at https://publons.com/publon/10.1111/jeb.14011.

## Supporting information

Appendix S1

## Data Availability

The raw data and R code for analysis are available on Dryad (https://doi.org/10.5061/dryad.bk3j9kdf9).
