# Supplementary material for: Negotiating mutualism: A locus for exploitation by rhizobia has a broad effect size distribution and context‐dependent effects on legume hosts
Source: J Evol Biol. 2022 May 4;35(6):844–54. doi: 10.1111/jeb.14011 (PMC9325427; doi:10.1111/jeb.14011)
Supplement: Supplementary file 1 — Appendix S1 [file JEB-35-844-s001.docx]

**Supplementary Information for “Negotiating mutualism: a locus for exploitation by rhizobia has a broad effect size distribution and context-dependent effects on legume hosts”**

**Table S1.** Methodological details for each of the greenhouse experiments.

| ***Method*** | ***Knockout Experiment*** | ***G*** × ***G Knockout Experiment*** |
| --- | --- | --- |
| Scarifying seeds | 9 May 2018 | 3 June 2019 |
| Sterilizing seeds | 14 May 2018 | 10 June 2019 |
| Planting seeds | 16 May 2018 | 11 June 2019 |
| Streaking strains from freezer stock | 14 May 2018 | 10 June 2019 |
| PCR check for *hrrP* | 18 and 21 May 2018 | 11 March 2019 |
| Inoculating 1mL cultures | 25 May 2018 | 21 June 2019 |
| Inoculating large cultures | 28 May 2018 | 24 June 2019 |
| Measuring OD_600_ of rhizobium cultures | 30 May 2018; using plate reader | 26 June 2019; using Nanodrop |
| Pelleting and resuspending cells | Pelleted cultures at 7000 rpm / 7 min; resuspended pellets in 0.1x TYB to final concentration | 5mL cultures sat in fridge overnight before inoculum prep; pelleted cultures at 4000rpm / 7 min; resuspended pellets in 0.1x TYB to final concentration |
| Inoculation date | 30 May 2018 | 27 June 2019 |
| Inoculation amount per plant | 900uL; 2 x 10^6^ CFU total | 450uL; 2 x 10^6^ CFU total |
| Date of adding sterilized sand to pots | 4 June 2019 | 1 July 2019 |
| Fertilizer addition dates | 6 June 2018  13 June 2018  20 June 2018  27 June 2018  3 July 2018 | 3 July 2019  10 July 2019  17 July 2019  24 July 2019  31 July 2019 |
| Harvest dates | 9-16 July 2018 (40-47 days post inoculation) | 5-9 Aug 2019 (39-43 days post inoculation) |
| Data collected from each plant | *Before harvest:*   - Leaf count (6 July; 37 days post inoculation)   *At harvest:*   - Shoot mass - Nodule count - CFU per average nodule | *At harvest:*   - Shoot mass - Nodule count - CFU per largest nodule |

**Table S2.** Genetic distance matrix for partial *hrrP* sequences from the 12 *hrrP*+ *E. medicae* strains used in our study plus the B800 *hrrP* allele (Price et al. 2015). The number of base substitutions per site is shown for each pair of strains. Cell color intensity scales with number of base substitutions per site. Sequences were downloaded from Genbank (see Table 1 for accession numbers) and analyzed with MEGA 7.0 (Kumar et al. 2016). Positions with gaps or missing data were deleted before analysis, with 190 nt included in the final distance matrix.

|  | AZN131 | AZN234 | DCR341 | PEA63 | PEA143 | RTM196 | RTM371 | RTM372 | RTM373 | RTM376 | STA354 | STA355 | B800 |
| --- | --- | --- | --- | --- | --- | --- | --- | --- | --- | --- | --- | --- | --- |
| AZN131 |  |  |  |  |  |  |  |  |  |  |  |  |  |
| AZN234 | 0.005 |  |  |  |  |  |  |  |  |  |  |  |  |
| DCR341 | 0 | 0.005 |  |  |  |  |  |  |  |  |  |  |  |
| PEA63 | 0.016 | 0.021 | 0.016 |  |  |  |  |  |  |  |  |  |  |
| PEA143 | 0 | 0.005 | 0 | 0.016 |  |  |  |  |  |  |  |  |  |
| RTM196 | 0.095 | 0.101 | 0.095 | 0.108 | 0.095 |  |  |  |  |  |  |  |  |
| RTM371 | 0.095 | 0.101 | 0.095 | 0.108 | 0.095 | 0 |  |  |  |  |  |  |  |
| RTM372 | 0.095 | 0.101 | 0.095 | 0.108 | 0.095 | 0 | 0 |  |  |  |  |  |  |
| RTM373 | 0.095 | 0.101 | 0.095 | 0.108 | 0.095 | 0 | 0 | 0 |  |  |  |  |  |
| RTM376 | 0.095 | 0.101 | 0.095 | 0.108 | 0.095 | 0 | 0 | 0 | 0 |  |  |  |  |
| STA354 | 0.050 | 0.056 | 0.050 | 0.062 | 0.050 | 0.075 | 0.075 | 0.075 | 0.075 | 0.075 |  |  |  |
| STA355 | 0.190 | 0.198 | 0.190 | 0.199 | 0.190 | 0.188 | 0.188 | 0.188 | 0.188 | 0.188 | 0.225 |  |  |
| B800 | 0.081 | 0.087 | 0.081 | 0.088 | 0.081 | 0.086 | 0.086 | 0.086 | 0.086 | 0.086 | 0.108 | 0.128 |  |

**Table S3. Pearson correlation coefficients for pairwise correlations among response variables for plants with nodules in the Knockout Experiment (n = 387) and G** × **G Knockout Experiment (n = 259).** *** P < 0.0001, ** P < 0.001, *, P < 0.05.

| ***Response 1*** | ***Response 2*** | ***Knockout Experiment*** | ***G × G Knockout Experiment*** |
| --- | --- | --- | --- |
| Leaf count | Shoot mass (g) | 0.83*** | na |
| Leaf count | Shoot per nodule | 0.30*** | na |
| Leaf count | Nodule count | 0.18** | na |
| Leaf count | Log(CFU per nodule) | 0.20*** | na |
| Shoot mass (g) | Shoot per nodule | 0.31*** | 0.55*** |
| Shoot mass (g) | Nodule count | 0.34*** | 0.36*** |
| Shoot mass (g) | Log(CFU per nodule) | 0.14* | 0.02 |
| Shoot per nodule | Nodule count | -0.61*** | -0.43*** |
| Shoot per nodule | Log(CFU per nodule) | 0.20*** | -0.10 |
| Nodule count | Log(CFU per nodule) | -0.13* | 0.08 |


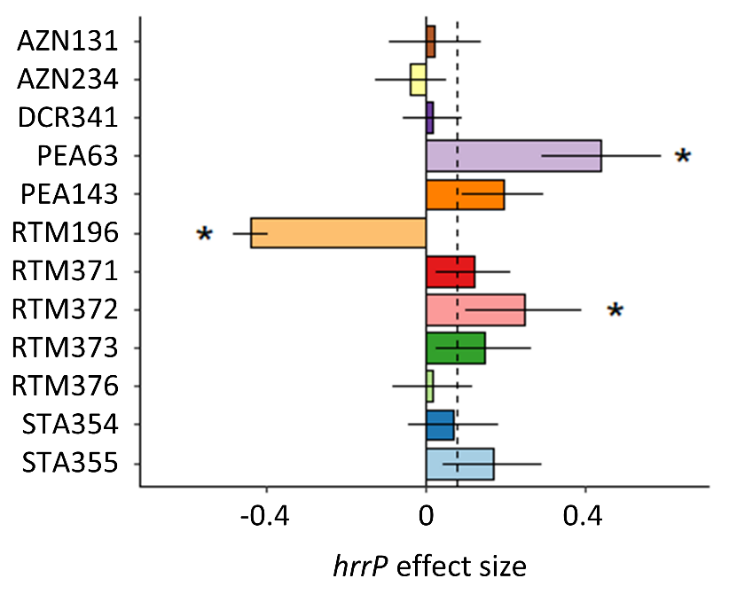


**Fig S1. Different *E. medicae* strains have contrasting effects of *hrrP* on leaf count**. *hrrP* effect size indicates the proportional change in a phenotype value due to the presence of *hrrP*, using comparisons of wildtype *hrrP*+ and knockout *hrrP*- mutant strains*.* Effect size was measured in the Knockout Experiment (Model 1)*.* Asterisks indicate parameter estimates of *hrrP* effect size for which the 95% confidence interval did not include zero. The dashed vertical line indicates the mean *hrrP* effect size across all 12 *hrrP* alleles. Bars represent +/- 1 SE.

**
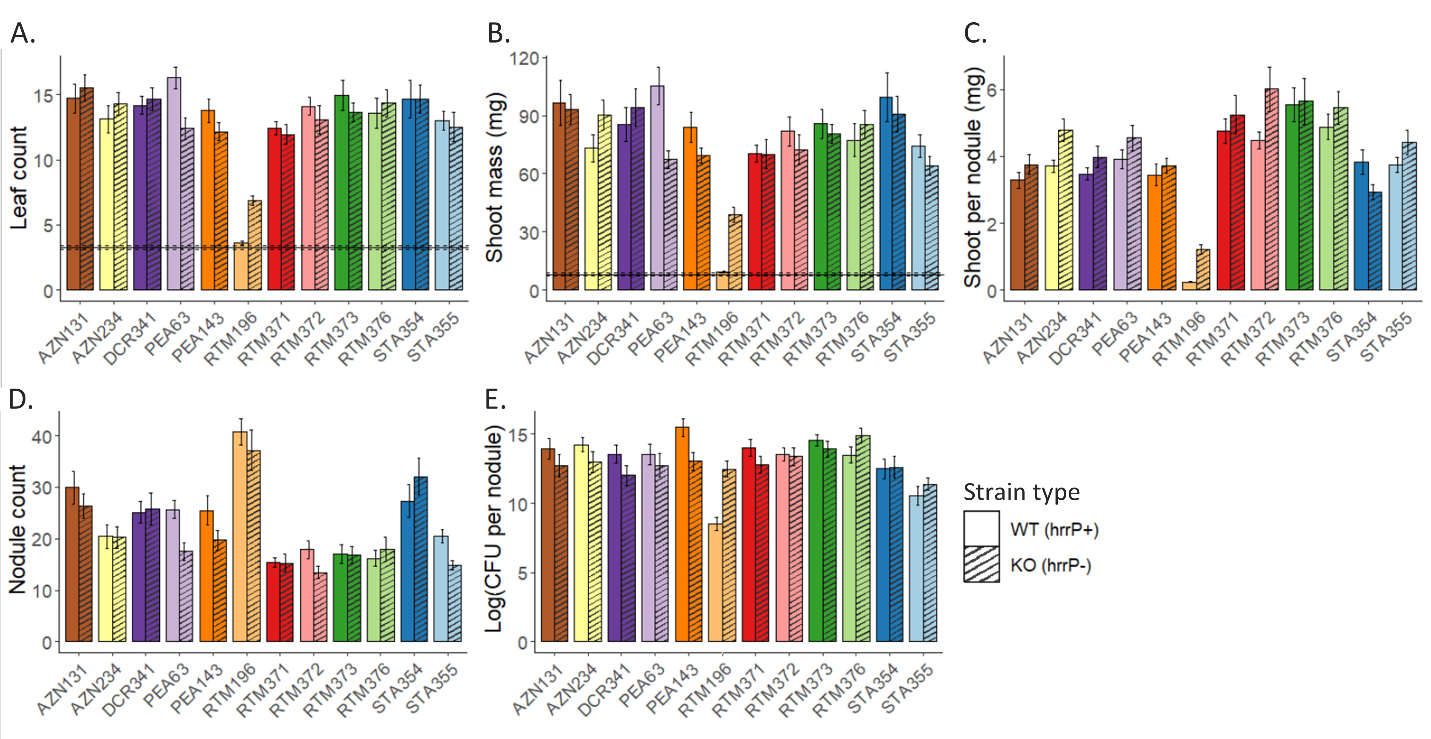
**

**Fig S2. Mean trait values of *M. polymorpha* RTM plants inoculated with wildtype (*hrrP*+) or knockout (*hrrP*-) *E. medicae* strains the Knockout Experiment.** From these raw data, *hrrP* effect size was calculated for each pair of wildtype and knockout plants as (Trait_WT_ – Trait_KO_) / Trait_KO_. Bars show means +/- 1 standard error. Bars are colored by *E. medicae* strain as in the main text. For panels A and B, horizontal lines indicate mean +/- 1 standard error trait values for uninoculated control plants.


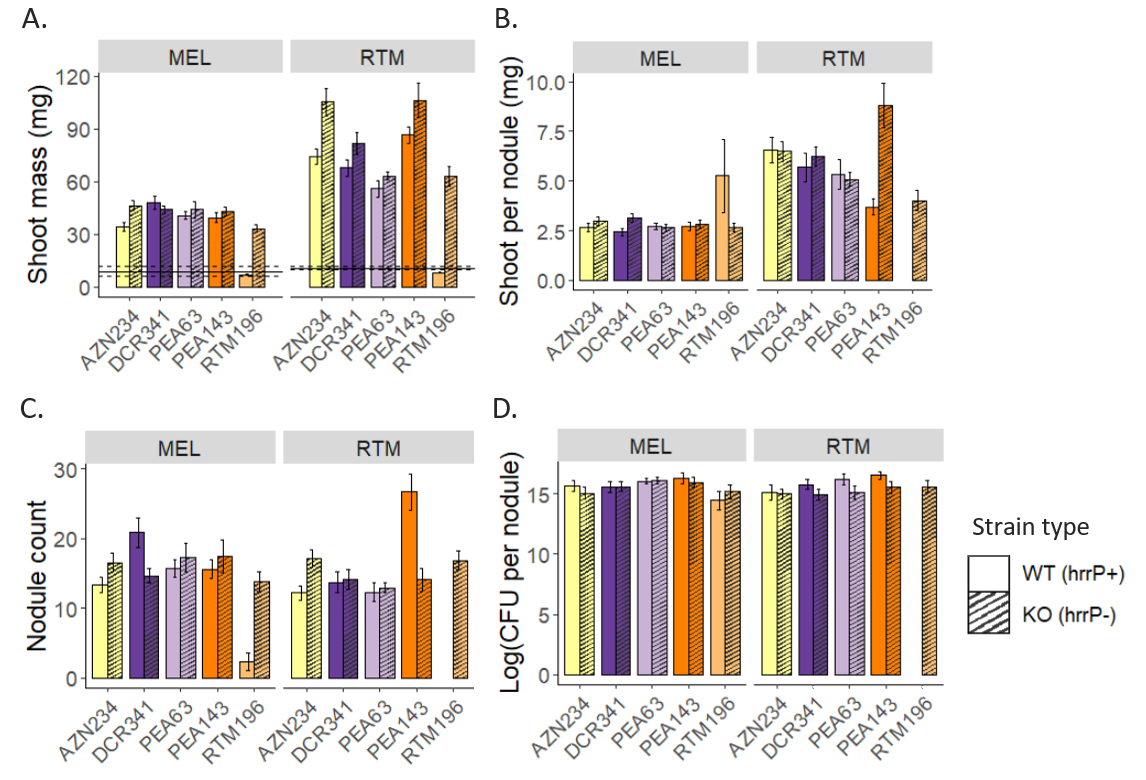


**Fig S3. Mean trait values of *M. polymorpha* MEL and RTM plants inoculated with wildtype (*hrrP*+) or knockout (*hrrP*-) *E. medicae* strains the G *×* G Knockout Experiment.** From these raw data, *hrrP* effect size was calculated for each pair of wildtype and knockout plants as (Trait_WT_ – Trait_KO_) / Trait_KO_. Bars show means +/- 1 standard error. Bars are colored by *E. medicae* strain as in the main text. For panel A, horizontal lines indicate mean +/- 1 standard error trait values for uninoculated control plants.

**References**

Kumar, S., G. Stecher, and K. Tamura. 2016. MEGA7: Molecular Evolutionary Genetics Analysis version 7.0 for bigger datasets. Molecular Biology and Evolution 33:1870-1874.

Price, P. A., H. R. Tanner, B. A. Dillon, M. Shabab, G. C. Walker, and J. S. Griffitts. 2015. Rhizobial peptidase HrrP cleaves host-encoded signaling peptides and mediates symbiotic compatibility. Proceedings of the National Academy of Sciences 112:15244-15249.
